# Endovascular treatment for sphenoidal region dural arteriovenous fistula

**DOI:** 10.3389/fneur.2024.1348178

**Published:** 2024-01-31

**Authors:** Jinlu Yu

**Affiliations:** Department of Neurosurgery, The First Hospital of Jilin University, Changchun, Jilin, China

**Keywords:** sphenoid region, dural arteriovenous fistula, endovascular treatment, prognosis, complication

## Abstract

Sphenoidal region dural arteriovenous fistulas (DAVFs) are rare. Endovascular treatment (EVT) is an effective treatment approach. However, understanding and performing EVT for sphenoidal region DAVFs are difficult and challenging. Therefore, we performed a review to explore this issue further. In this review, we discuss the dural feeders and venous structures of the sphenoidal region, the angioarchitecture of sphenoidal region DAVFs, the role and principle of EVT, various EVT techniques, and the prognosis and complications associated with EVT. We found that various EVT techniques, including transarterial embolization (TAE), retrograde transvenous embolization (TVE), and direct puncture EVT, can be used to treat sphenoidal region DAVFs. TAE represents the most commonly utilized approach. TVE and direct puncture EVT should be limited to highly selective cases. EVT must penetrate the fistula and very proximal venous recipient pouch with a liquid embolic agent or coil the fistula point to have a complete curative effect. Successful EVT can lead to the obliteration of sphenoidal region DAVFs and a good clinical outcome. However, these complications cannot be neglected.

## 1 Introduction

Sphenoidal region dural arteriovenous fistula (DAVF) is a rare aberrant connection between meningeal branches from the external carotid artery (ECA) and the internal carotid artery (ICA) and venous structures of the sphenoid region (1–3). A DAVF may be spontaneous, traumatic, or iatrogenic (4–7). When a DAVF results in venous hypertension, patients experience brain edema and hemorrhage. Treatment for DAVFs, including open surgery, endovascular treatment (EVT), and stereotactic radiosurgery, is necessary (8, 9).

Currently, EVT is an effective alternative for treating these lesions. Because of complex venous drainage in the sphenoidal region, sphenoidal region DAVFs have complex angioarchitectures, which make EVT challenging (1). To date, insufficient knowledge has been produced regarding EVT for sphenoidal region DAVFs. Therefore, we performed a review to explore this issue further and provide some illustrative cases.

## 2 Dural feeders and venous structures of the sphenoidal region

**Figure 1:**
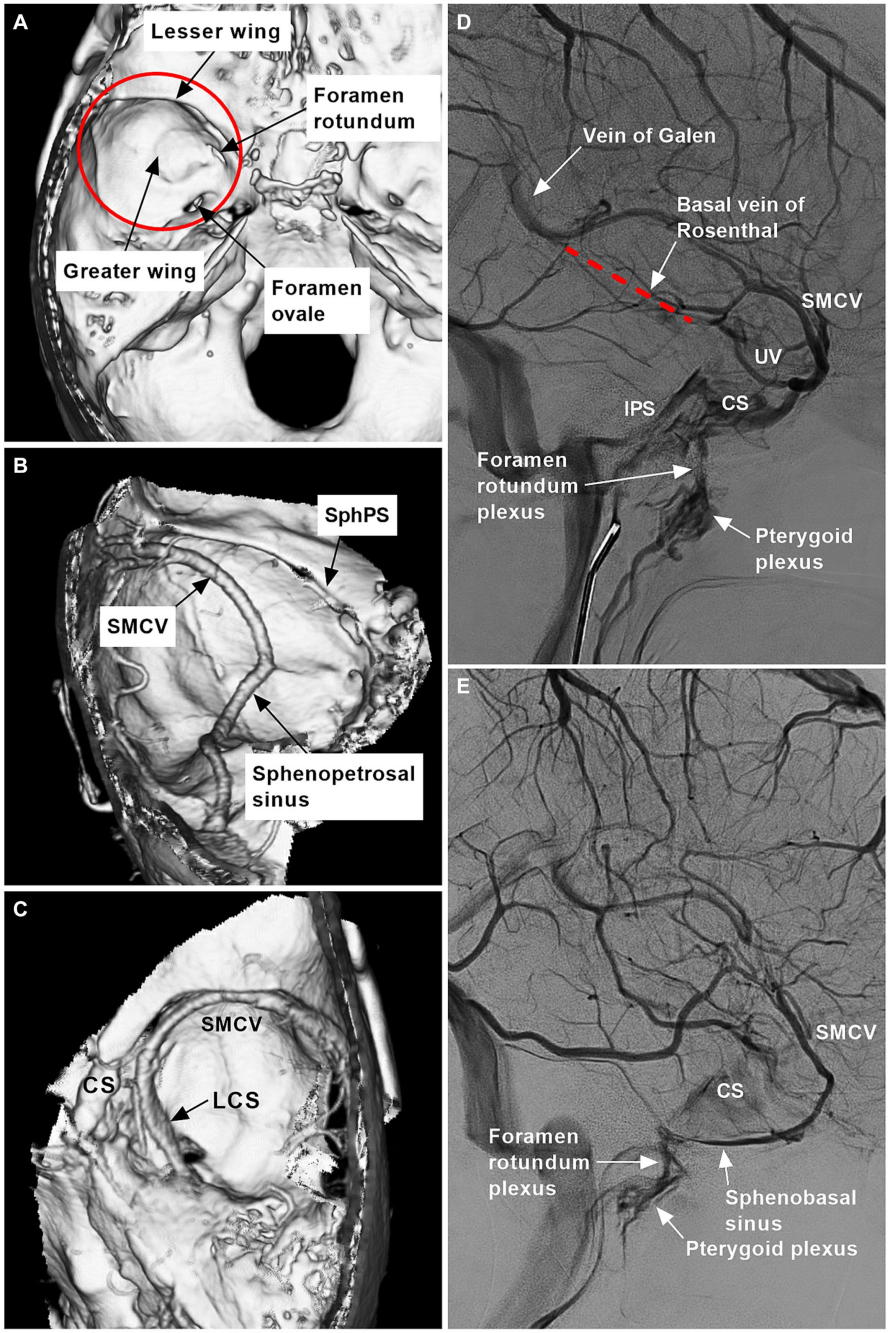
Range and venous structures of the sphenoidal region. **(A)** Reconstructive three-dimensional CT image showing the sphenoidal region (red circle) and bony structures (arrows). **(B)** CTA image showing the SphPS, SMCV, and sphenopetrosal sinus (arrows). **(C)** CTA image showing the LCS (arrow), CS, and SMCV. **(D)** DSA image showing the vein of Galen, the basal vein of Rosenthal (red dotted line), the foramen rotundum plexus, the pterygoid plexus (arrows), the IPS, the CS, the SMCV, and the UV. **(E)** DSA showing the foramen rotundum plexus, pterygoid plexus and sphenobasal sinus (arrows), CS, and SMCV. CS, cavernous sinus; CT, computed tomography; CTA, CT angiography; DSA, digital subtraction angiography; IPS, inferior petrous sinus; LCS, laterocavernous sinus; SMCV, superior middle cerebral vein; SphPS, sphenoparietal sinus; UV, uncal vein.

The sphenoidal region is located in the central cranial fossa and is composed of the greater and lesser wings (Figure 1A). The meningeal branches from the ICA and ECA supply the dura of the sphenoidal region. Several dural sinuses and bridging and diploic veins can be involved in venous drainage of the sphenoidal region (Figures 1B–E).

### 2.1 Dural feeders

Normal arterial contributions to the dura of the sphenoid region arise from ECA branches, including the middle meningeal artery (MMA), accessory meningeal artery (AMA), foramen rotundum artery, and middle deep temporal artery; from cavernous ICA branches, including the inferolateral trunk (ILT) and meningohypophyseal trunk (MHT); and from recurrent meningeal branches of the ophthalmic artery (OphA) (1, 10).

### 2.2 Dangerous anastomosis

Dural feeders of the sphenoid region can anastomose together, forming a dangerous anastomosis. The OphA is the core of dangerous anastomosis (11). The anterior convexity branch of the MMA runs along the greater wing of the sphenoid to supply the dura and communicates with the lacrimal branch of the OphA via the meningolacrimal artery. This artery can anastomose to the proximal OphA or ILT via the recurrent meningeal artery. The petrosal branch of the MMA can anastomose to the branches of the ILT and MHT (12, 13). The intracranial branch of the AMA passes through the foramen ovale or the foramen Vesalius to anastomose to the ILT and MHT at the cranial base (14).

### 2.3 Venous structures

The venous structures of the sphenoidal region include the cavernous sinus (CS), sphenoparietal sinus (SphPS), paracavernous sinus (PCS; sphenobasal and sphenopetrosal sinuses), laterocavernous sinus (LCS), superior middle cerebral vein (SMCV), uncal vein (UV) and middle meningeal vein (MMV). These venous structures connect with each other because, embryologically, there are two anastomosed venous systems, the prootic sinus and primitive tentorial sinus systems. The former develops into the ophthalmic vein and CS, and the latter develops into the SMCV to connect to the lateral sinus (15).

#### 2.3.1 SphPS and PCS

SphPS begins at the lateral tip of the sphenoid lesser wing and courses in the dura mater just below the sphenoid ridge. The SphPS may communicate indirectly with the superior sagittal sinus via the MMV (16, 17). SphPSs may receive tributaries from vessels, including the SMCV and, occasionally, deep Sylvian veins, the MMV, diploic veins, and small dural veins (18, 19). The SphPS may empty into the anterior part of the CS. SphPSs can join the sphenoidal emissary vein into the pterygoid plexus through the foramen rotundum, where it is referred to as the sphenobasal sinus. SphPSs can join the superior petrosal sinus (SPS) or lateral sinus, where they are referred to as the sphenopetrosal sinus (16, 18, 20, 21). Sphenobasal and sphenopetrosal sinuses are located in the middle fossa base along the sphenoid greater wing, laterally and medially, respectively (Figures 1B,E).

#### 2.3.2 LCS, SMCV, UV, and MMV

*The* LCS is a venous space located between two dural layers that forms the lateral wall of the CS (Figure 1C) (22, 23). The LCSs can be identified as contiguous to the SMCV drainage site. LCSs can drain into the lateral sinus via the SPS, into the pterygoid plexus via the venous plexus of the foramen rotundum, or into the posterior aspect of the CS via anastomosis (24, 25). The SMCV courses under the sphenoid lesser wing and can connect with the proximal SphPS or directly drain into the lateral aspect of the CS, into the LCS, or into the PCS following a more lateral trajectory (Figures 1B–E) (21).

The UV is a small deep vein and can drain into the CS, SMCV, LCS, or PCS (19). The UV can serve as an anastomotic channel between the CS and the basal vein of Rosenthal (Figure 1D). After leaving the anterior branch of the MMA at the lateral tip of the sphenoid lesser wing, the MMV can join the SphPS and drain into the CS or sphenoidal emissary vein. The MMV can also accompany the posterior branch of the MMA into the lateral sinus. Because the MMV has an intradiploic course, it can reach the orbital and anterior temporal diploic veins (16, 26).

## 3 Angioarchitecture of sphenoidal region DAVF

### 3.1 Classification

**Figure 2:**
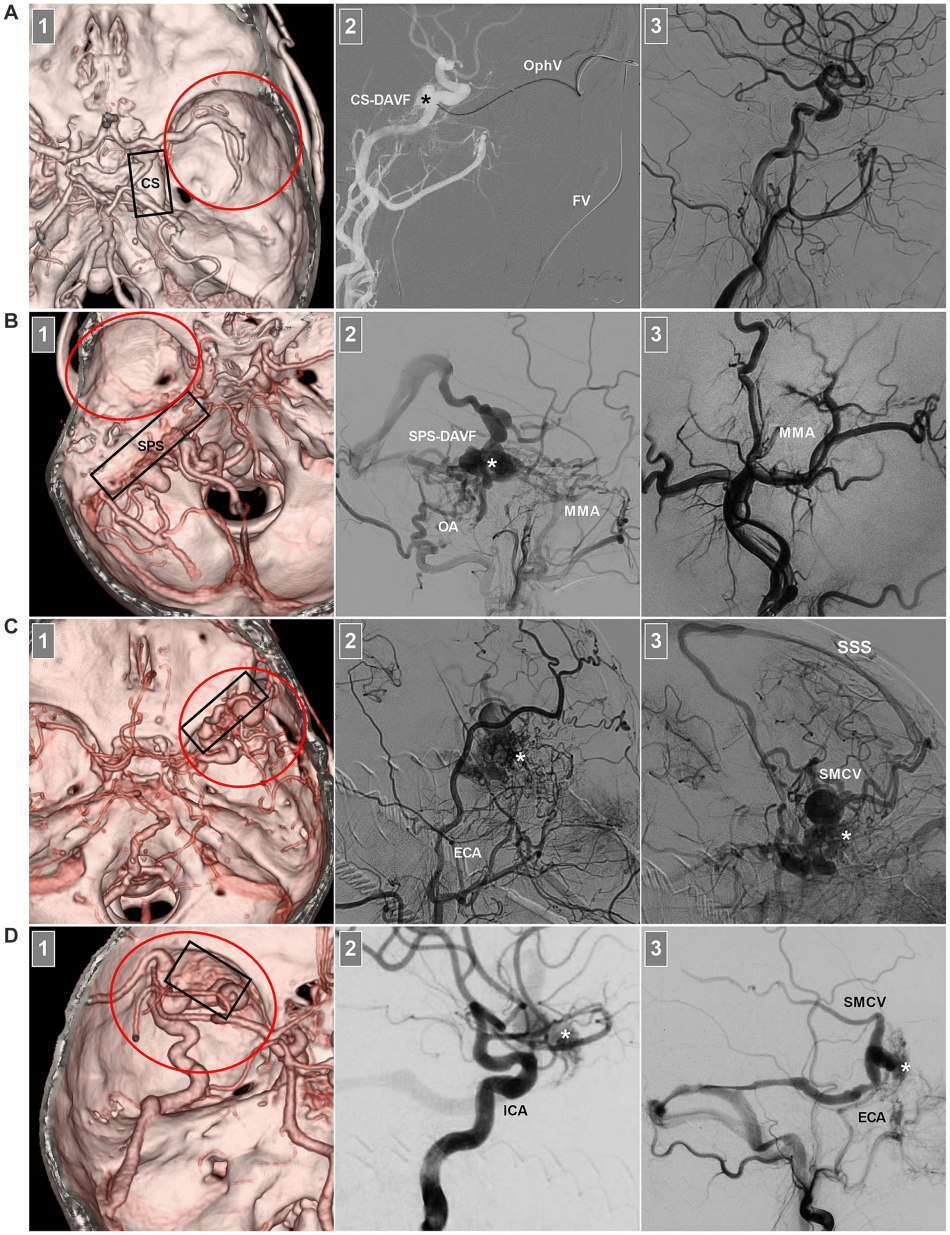
Middle cranial fossa DAVFs. **(A)** Number 1 panel: CTA image showing a CS-DAVF (frame) outside the sphenoidal region (red circle); Number 2 panel: Roadmap DSA image showing that the fistula (asterisk) was catheterized via the FV and superior OphV. Number 3 panel: DSA image showing that the fistula was obliterated. **(B)** Number 1 panel: CTA image showing an SPS-DAVF (frame) outside the sphenoidal region (red circle); Number 2 panel: DSA image showing the fistula (asterisk) supplied by the OA and MMA. Number 3 panel: DSA showing that the fistula was obliterated via the MMA. **(C)** Number 1 panel: CTA image showing a sphenoidal lesser wing DAVF (frame) in the sphenoidal region (red circle); Number 2 panel: DSA image showing the fistula (asterisk) supplied by numerous ECA branches; Number 3 panel: venous phase DSA image showing the fistula (asterisk) draining into the SMCV and cortical veins into the SSS. **(D)** Number 1 panel: CTA image showing a sphenoidal greater wing DAVF (frame) in the sphenoidal region (red circle); Number 2 panel: DSA image showing the fistula (asterisk) supplied by the ICA branch; Number 3 panel: DSA image showing the fistula (asterisk) supplied by the ECA draining into the SMCV and transverse sinus via the sphenopetrosal sinus. CS, cavernous sinus; CTA, computed tomography angiography; DAVF, dural arteriovenous fistula; DSA, digital subtraction angiography; ECA, external carotid artery; FV, facial vein; MMA, middle meningeal artery; OA, occipital artery; OphV, ophthalmic vein; SMCV, superior middle cerebral vein; SPS, superior petrous sinus; SSS, superior sagittal sinus.

Sphenoidal region DAVFs are middle cranial fossa DAVFs; however, middle cranial fossa DAVFs still include CS and SPS-DAVFs (Figures 2A,B) and do not belong to the sphenoidal region (5). Sphenoidal region DAVFs are composed of sphenoid lesser or greater wing DAVFs (27–29). Sphenoid lesser wing DAVFs occur along the sphenoid ridge from the medially anterior clinoid process to the lateral pterion (Figure 2C) (5, 30–32). Sphenoid greater wing DAVFs include PCS and LCS DAVFs (Figure 2D) (24, 33, 34). PCS DAVFs include sphenopetrosal and sphenobasal DAVFs (35).

In addition to the anatomical location, there are other classifications for sphenoidal region DAVFs. These tumors can also be divided into fine-network and single-channel types based on the fistulous structure, into high-flow and low-flow types based on the shunting blood flow, and into dural type and intraosseous diploic types based on the remodeling or erosion of the sphenoid bone (28, 29, 36–38).

### 3.2 Arterial supply

**Figure 3:**
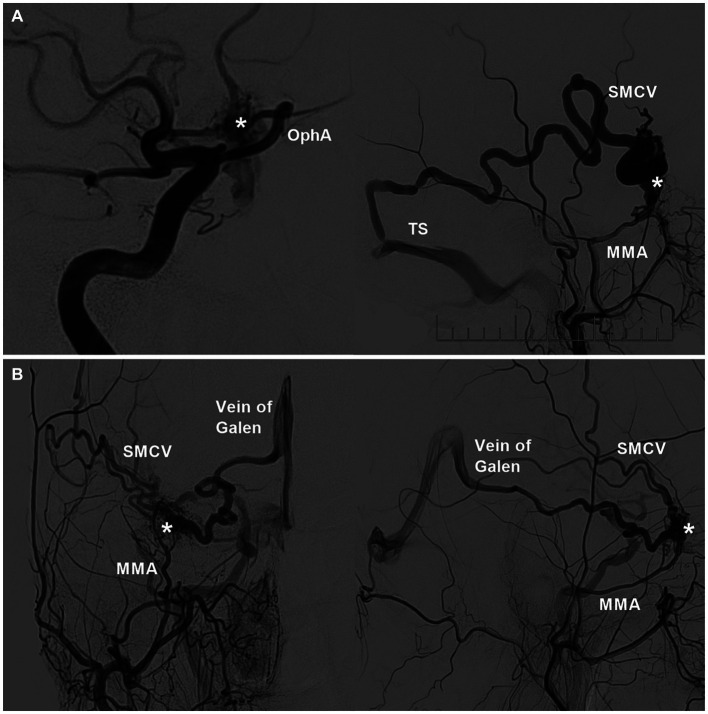
Feeding arteries and venous drainages of sphenoidal region DAVFs. **(A)** Left: DSA image showing the DAVF (asterisk) supplied by the recurrent meningeal branch of the OphA; right: DSA image showing the DAVF (asterisk) supplied by the MMA and drained into the SMCA and then into the TS. **(B)** DSA image of the anterior posterior view (left) and lateral view (right) showing the DAVF (asterisks) supplied by the MMA and drained into the SMCA and basal vein of Rosenthal into the vein of Galen. DAVF, dural arteriovenous fistula; DSA, digital subtraction angiography; MMA, middle meningeal artery; OphA, ophthalmic artery; SMCV, superior middle cerebral vein; TS, transverse sinus.

Although sphenoid region DAVFs vary in location, they share similar feeding arteries (35). The most important feeding arteries for these lesions arose from the MMA and AMA of the ECA. The meningeal branches of the ICA included the ILT and the recurrent meningeal branch from the OphA (Figure 3A) (30, 32, 36). Sphenoid region DAVFs can recruit additional feeding arteries, such as the MHT, ethmoidal branch of the OphA, artery of the foramen rotundum, deep temporal artery, meningoorbital artery, superficial temporal artery, anterior auricular artery, and ascending pharyngeal artery (1–3, 5, 9, 30, 33, 39–41). Even feeding arteries from the contralateral ICA and ECA can be recruited (40, 42).

### 3.3 Venous drainage

Sphenoid region DAVFs can drain into the SphPS, CS, PCS, LCS, SMCA, ophthalmic vein (OphV), and diploic or transosseous emissary veins (5, 8, 28, 33, 35, 43–45). Sphenoid intraosseous diploic DAVFs may drain via the diploic vein or MMV (29). These venous drainage tubes must then be used to find adjacent outlets. On rare occasions, retrograde filling into the basal vein of Rosenthal and vein of Galen is observed (Figure 3B) (17, 39, 40, 46, 47). The basal vein of Rosenthal anastomosis to the lateral mesencephalic vein may result in drainage into the venous system of the brainstem (47). Venous hypertension often results in ectasias and aneurysms in the arterialized draining vein (8, 31, 36, 48). Therefore, Borden type III and Cognard type IV lesions are common (49, 50).

## 4 Clinical presentation

The venous drainage pattern of sphenoid region DAVFs determines clinical presentation (2, 44). DAVFs of the sphenoid greater wing, including PCS and LCS DAVFs, often drain into the SMCV, resulting in cortical venous hypertension with a greater frequency of varices (1). Therefore, subarachnoid and intraparenchymal hemorrhages are common in DAVFs of the sphenoid greater wing due to fistula or draining vein rupture (1, 33, 47).

DAVFs of the sphenoid lesser wing often exhibit epidural venous drainage into the CS, leading to eye symptoms similar to those of a CS DAVF (2, 3, 5, 9, 27). However, due to venous drainage variants and occlusive or stenotic drainage into the CS, DAVFs cannot drain into the CS but can retrograde into the cortical vein, the basal vein of Rosenthal, or the MMV, resulting in symptoms such as DAVFs of the sphenoid greater wing, also presenting with subarachnoid and intraparenchymal hemorrhages (2, 9, 47, 51).

## 5 EVT role and principles

### 5.1 Role and status of EVT

Currently, EVT for sphenoidal region DAVFs is becoming an effective option due to progress in EVT products and techniques. However, the use of EVT for these complex lesions is challenging due to the rarity and lack of accessible transarterial or transvenous routes. Not all lesions can be cured by EVT. In Shi et al.’s report of 11 sphenoidal region DAVFs, only 9 lesions were cured by EVT (1). In Shimizu et al.’s review of 15 sphenoid wing DAVFs, only 8 lesions were cured by EVT (52).

**Figure 4:**
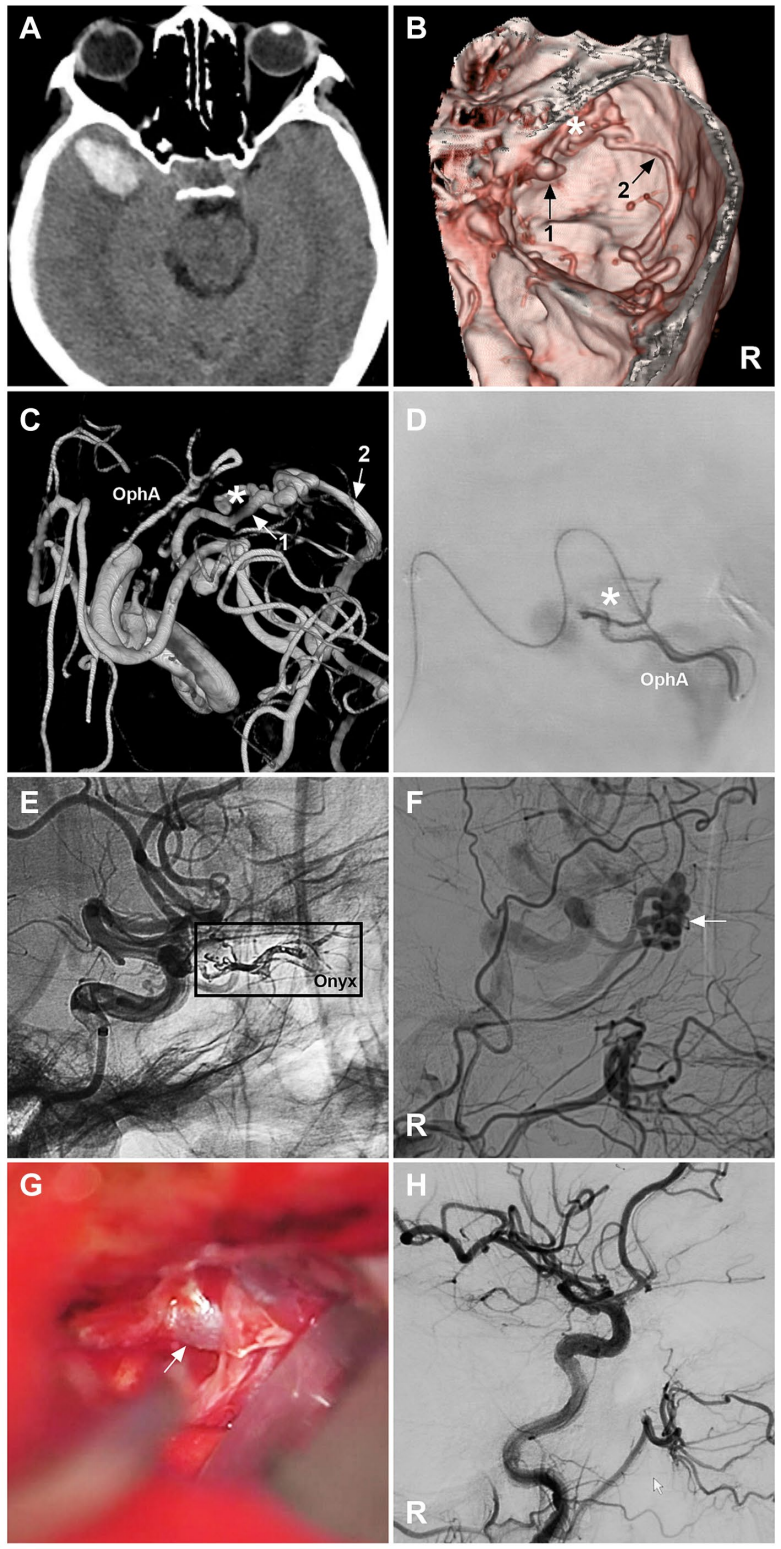
Open surgery after incomplete EVT for a sphenoidal region DAVF. **(A)** CT image showing a right temporal lobe hematoma. **(B)** CTA image showing a right sphenoidal lesser wing DAVF (asterisk); numbers 1 and 2 with arrows indicate venous drainage. **(C)** Three-dimensional DSA image showing that the DAVF was supplied by the recurrent meningeal branch of the OphA; the numbers 1 and 2 with arrows indicate venous drainage. **(D)** Selective angiography showing the DAVF (asterisk). **(E)** Unsubtracted DSA showing Onyx casting (frame) via the recurrent meningeal branch of the OphA. **(F)** DSA image showing the residual DAVF (arrow). **(G)** Intraoperative image of an open surgery showing the arterialized draining vein (arrow); then, the vein was coagulated and cut. **(H)** Immediate DSA showing that the DAVF was obliterated. CT, computed tomography; CTA, computed tomography angiography; DAVF, dural arteriovenous fistula; DSA, digital subtraction angiography; EVT, endovascular treatment; R, right; OphA, ophthalmic artery. After EVT, the patient suffered right eye blindness.

When EVT fails, open surgery in a hybrid operating room can be performed (Figure 4). DAVFs can be cured by coagulation of feeding arterial networks, disconnection of venous drainage close to the fistula, and resection of the lesion (1, 8, 9, 46, 53). For lesions at high risk of surgery and EVT, radiation is an alternative; however, the main disadvantage of radiation is the delayed effect of this treatment modality.

### 5.2 EVT aims and principles

For EVT, it is important to recognize the venous draining pattern and the anatomy of sphenoidal region DAVFs (44). For EVT to have a curative effect, the fistula point must be coiled completely or the fistula and most proximal venous recipient pouch must be penetrated with a liquid embolic agent. Any recruited draining veins were obliterated. Only occlusion of the feeding arteries allows the recruitment of the feeding arteries of the DAVF to recanalize. In addition, incomplete occlusion of DAVFs may divert blood flow into cortical or deep veins.

## 6 Various EVT techniques

Various EVT techniques, including transarterial embolization (TAE), retrograde transvenous embolization (TVE), and direct puncture EVT, can be used to treat sphenoidal region DAVFs. These approaches may be used individually or in combination (27). Among these techniques, TAE represents the most commonly utilized approach.

### 6.1 Embolic agents

For EVT of sphenoidal region DAVFs, coil and liquid embolic agents can be used. The liquid embolic systems used included Onyx (Medtronic, Irvine, California, United States), Squid (Balt Extrusion, Montmorency, France), and PHIL (MicroVention, Inc., Aliso Viejo, CA, United States), as well as “glue,” such as N-butyl cyanoacrylate (NBCA; Cordis, Miami Lakes, FL, United States). Onyx was more popular than other liquid embolic agents for treating DAVFs.

For DAVFs with a venous recipient pouch, if the fistula connection or venous recipient pouch can be catheterized, only coiling may be sufficient (4, 36, 43). Coils can be delivered by microcatheters (0.014 in.) of Echelon-10 (Medtronic, Irvine, California, United States), SL-10 (Stryker Neurovascular, Fremont, CA, United States), and Headway-17 (MicroVention, Inc., Aliso Viejo, CA, United States). In addition, if coiling is insufficient, after coils are detached into a high-flow shunt to better visualize and control flow, definitive occlusion with additional liquid embolic agents can be performed (1). In addition, coils can also be used to create a plug to lock the working microcatheter via the “pressure cooker” technique.

Onyx is a nonadhesive embolic agent that can significantly increase the efficiency of penetrating fistulas. The reflux-hold-reinjection technique allows deep and controlled Onyx penetration into the fistula and proximal draining vein (1). Onyx had Onyx-34 and Onyx-18 types. The Onyx can be cast mainly by a Marathon microcatheter (Medtronic, Minneapolis, MI, United States), an Apollo microcatheter with a detachable tip (Medtronic, Minneapolis, MI, United States), or an Echelon-10 microcatheter (Medtronic, Minneapolis, MI, United States). For TAE, Onyx-18 was preferred. For TVE, Onyx-34 can be chosen. Onyx-34 can also be used to create a plug in the ‘pressure cooker’ technique (28).

NBCA can be used as an adhesive glue. A less tortuous feeder with a smaller caliber and slower flow should be selected to allow a microcatheter to be wedged into position. After the NBCA penetrates the fistula, sphenoidal region DAVFs can be obliterated (29, 39, 40, 54).

The squid is a novel nonadhesive liquid embolic agent similar to Onyx (55). Two major innovations in comparison with Onyx are that squid has a very small grain size of admixed radiopaque tantalum powder and an additional extra-low viscous formulation, which leads to a slowdown of this sedimentation process, potentially extending the duration of adequate visibility and lowering the risk of microcatheter occlusion. In a recent report, squid was found to be a safe and effective liquid embolic agent for the treatment of high-grade DAVFs (56). It was appropriate to use this technique to embolize DAVFs in the sphenoid region.

PHIL is a nonadhesive agent comprising a copolymer dissolved in dimethyl sulfoxide. An iodine component is chemically bonded to the copolymer to provide radiopacity for fluoroscopic visualization. The potential advantages of PHIL include good forward flow with less reflux, together with the capability for repeated injections allowing for controlled target embolization. In Leyon et al.’s report of the use of the PHIL in treating cranial and spinal DAVFs, the PHIL was proven to be an excellent alternative embolic material (57). It can be used to embolize DAVFs in the sphenoid region.

### 6.2 TAE

**Figure 5:**
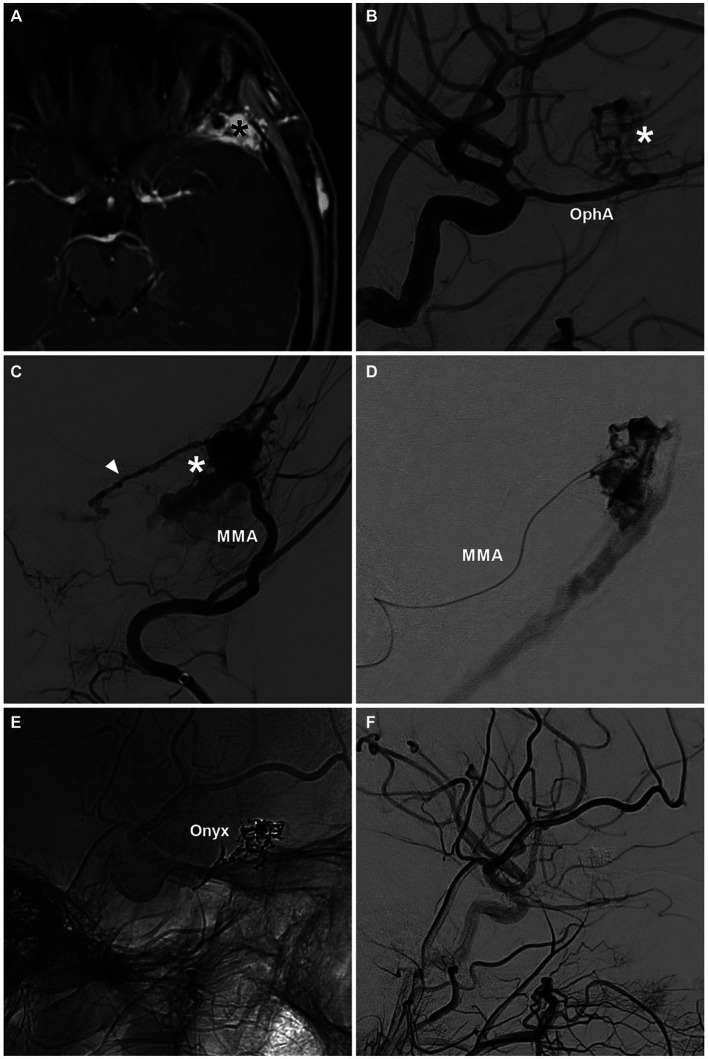
Complete EVT via the MMA for a sphenoidal region DAVF. **(A)** MRI image showing a high signal (asterisk) at the left sphenoidal ridge. **(B)** DSA image showing the DAVF (asterisk) supplied by the recurrent meningeal branch of the OphA. **(C)** DSA showing a sphenoidal ridge DAVF (asterisk) supplied by the MMA. The arrowhead indicates the anastomosis with the recurrent meningeal branch of the OphA. **(D)** Selective angiography of the MMA showing the DAVF. **(E)** Unsubtracted DSA showing the Onyx casting. **(F)** Post-EVT DSA showing that the DAVF was obliterated. DAVF, dural arteriovenous fistula; DSA, digital subtraction angiography; EVT, endovascular treatment; MMA, middle meningeal artery; MRI, magnetic resonance imaging; OphA, ophthalmic artery.

For sphenoidal region DAVFs, the MMA or AMA can act as the gold arterial path of TAE (Figure 5). The most complete TAEs for sphenoidal region DAVFs via MMA and AMA can be found in reports by Rezende et al. (40), Unterhofer et al. (4), Fukuda et al. (39), Macdonald et al. (2), Yako et al. (29), Park et al. (54), Kandyba et al. (17), and Shimizu et al. (52). In addition, on rare occasions, complete TAE for sphenoidal region DAVFs can be achieved via the artery of the foramen rotundum (39, 58). During TAE via the MMA or AMA, other large feeding arteries can be occluded with coils or balloons to reduce blood flow and facilitate liquid embolic agent casting (39).

During TAE, the microcatheter may even have difficulty accessing the fistula point due to the tortuous arterial approach. In this circumstance, retrograde reflux of the liquid embolic agent was often unavoidable. In addition, due to the presence of concurrent blood flow in DAVFs, liquid embolic agents can be prevented from penetrating fistulas. The “pressure cooker” technique was useful for preventing retrograde reflux of the liquid embolic agent and for driving the liquid embolic agent toward the fistula. A Scepter Mini balloon microcatheter (Microvention, Aliso Viejo, CA) can be used to conveniently establish the “pressure cooker” effect (59). However, the balloon microcatheter may be too stiff to close the fistula.

The “pressure cooker” technique can be established by using double microcatheters. Before performing liquid embolic agent casting using a working microcatheter, a plug made by another microcatheter can be established to occlude the distal feeding artery to prevent reflux of the liquid embolic agent. Working microcatheters may use Marathon or Apollo microcatheters. When using an Apollo microcatheter, the plug should be distal to its detachment point to facilitate microcatheter removal (1, 17, 33).

When the patient is wearing a casing liquid embolic agent via the “pressure cooker” technique, the uninvolved normal branch of the MMA can be coiled in advance to avoid liquid embolic agent entering into dangerous anastomosis into the ICA or OphA (17). In addition, to avoid inadvertent liquid embolic agent reflux into the ICA, a protective balloon can be navigated into the cavernous ICA and temporarily inflated to block retrograde liquid embolic agent from entering the ICA (17, 39, 40).

### 6.3 TVE

TVE can be used for sphenoidal region DAVFs fed by multiple small arterial feeders or a very tortuous course of feeding arteries; however, there are patent venous pathways to access fistulas, such as the facial vein and OphV, inferior petrous sinus and CS, superior sagittal sinus and cortical bridging vein, pterygoid plexus, and vein of Galen and vein of Rosenthal (1, 6, 7, 42). However, TVE should be limited in highly selective cases.

For TVE, when the fistula was catheterized, only coiling the fistula connection or venous pouch was a good option. For instance, in Kim et al.’s (60) and Misaki et al.’s (15) reports, transvenous catheterization to SphPS DAVFs by an SL-10 microcatheter was performed via the facial vein and superior OphV, after which the DAVF was completely coiled. In Akamatsu et al.’s report, a PCS DAVF was catheterized by an SL-10 microcatheter via the superior sagittal sinus (SSS) and SMCV, and the DAVF was completely coiled (43). In San et al.’s report, an LCS DAVF was completely coiled through the pterygoid plexus via the external jugular vein (61).

When coiling is unsatisfactory, liquid embolic agents can be combined with each other (1). In Agnoletto et al.’s report, a SphPS DAVF was accessed by two Echelon-10 microcatheters via the inferior petrous sinus and CS; coils were deployed through a microcatheter to obstruct outflow and serve as a lattice, and Onyx was then injected at the fistulous point through another microcatheter with good filling of the fistulous connection (6).

For TVE, four-dimensional (4D) digital subtraction angiography (DSA) and three-dimensional (3D) fused images are important for planning EVT strategies. In Ishibashi et al.’s report, a DAVF in the sphenoid bone with a large venous pouch was completely coiled via the inferior petrous sinus, and the CS after a microcatheter in the venous pouch was identified by 4D-DSA and 3D fused images (42).

In addition to routine microcatheters for delivering coils, the Marathon microcatheter can be used; it has both flow-guided and guidewire-guided characteristics so that it is useful both for advancing through the tortuous drainer and for selecting the target cavity. It has a distal inner diameter of 0.013. The Marathon microcatheter is not designed to deliver traditional coils. However, it can be used in the delivery of certain coils, such as detached Axium Prime coils (Medtronic, Irvine, CA, United States), Kaneka ED extrasoft coils (Kaneka, Kanagawa, Japan), and barricade coils (Blockade Medical, Irvine, CA, United States) (62–64). In Fukuda et al.’s report, after coiling the large feeding arteries of a sphenoidal wing DAVF to result in flow reduction, the Marathon microcatheter was introduced via the superior sagittal sinus, vein of Trolard, and anterior temporo-basal vein into the venous side of the fistula, and Kaneka ED coils were used to complete successful obliteration (39).

**Figure 6:**
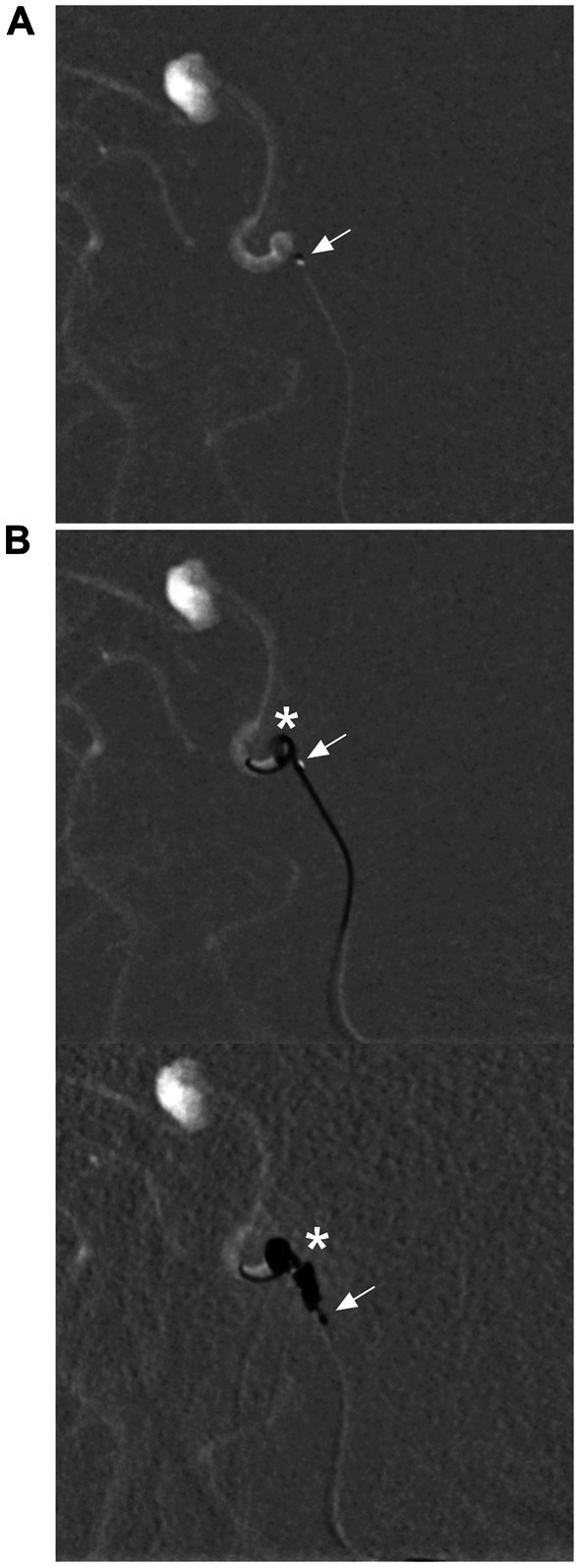
Delivery of coils via a Marathon microcatheter. **(A)** Roadmap DSA showing that the Marathon microcatheter (arrow) accessed the aneurysm. **(B)** Roadmap DSA showing that the Marathon microcatheter (arrows) was delivering Jasper®SS-10 coils (asterisks) (upper and lower). DSA, digital subtraction angiography.

Recently, there have been two types of available coils made in China that can be delivered via the Marathon microcatheter, the Visee coil (Visee Medical Devices Co., Ltd., Shandong, China) and the Jasper®SS-10 coil (Achieva Medical Co., Ltd., Shanghai, China), which provide additional options for coiling (Figure 6).

### 6.4 Direct puncture EVT

Sphenoidal region DAVFs can be embolized by direct puncture EVT. The puncture point can guide the draining vein directly or via a surgically created window or burr hole. In Cohen et al.’s report, after microsurgical exposure of the superior OphV allowed direct cannulation and catheterization, complete occlusion of a sphenoid greater wing DAVF was achieved by coiling (7). In White et al.’s report, after a burr hole was made over the diploic draining vein of an intraosseous DAVF of the sphenoid bone, coiling followed by Onyx casting was used to completely embolize the fistula and very proximal draining vein (45).

Direct puncture can be performed if the fistula is located in the bone or in the sinus of the epidural or intradural space. However, direct puncture should be guided by a navigation system. In the report by Nerva et al., after the TAE and TVE approaches failed to treat an intraosseous DAVF of the sphenoid bone and parasellar region, under Xper-computed tomography (CT) guidance from the DSA machine, a transfacial puncture was used to access the venous pouch. After embolization with coils and Onyx, the DAVFs within the sphenoid bone were embolized (28). In Dye et al.’s report, using a neuronavigation system with CT angiography images, the exact location of the fistula sac of a PCS DAVF was identified. Next, a spinal needle was used to guide the PCS, the Onyx was cast under fluoroscopic visualization, and the DAVF was resolved (41). In addition, endoscopic visualization was helpful for performing precise punctures. In Karas et al.’s report, under stereotactic guidance and endoscopic visualization, after direct puncture to the venous pouch, Onyx casting completely embolized a PCS DAVF (65).

## 7 Prognosis

Only occlusions of the distal feeding artery and fistula cannot cure sphenoidal region DAVFs; although angiography confirmed no residual DAVF, recanalization can occur (66). Occlusions of distal feeding arteries, fistula connections, and the very proximal segment of the draining vein can lead to obliteration of DAVFs and a good clinical outcome via TAE or TVE (1, 15, 17, 39, 40, 54, 58). For instance, in Lv et al.’s report, 7 LCS DAVFs were completely embolized, and good outcomes were achieved (67). In Shi et al.’s report of 11 sphenoidal region DAVFs, 9 successful EVTs were obtained, a good prognosis was achieved, and no recurrence was observed (1). In Shimizu et al.’s review, 8 sphenoid wing DAVFs were cured by 4 TAEs, 2 VE, and 2 TAEs plus TVE (52).

## 8 Complications

When a liquid embolic agent is cast via the MMA or AMA to treat a sphenoidal region DAVF, the embolic agent can migrate into the OphA, resulting in eye blindness because of potential embolization to the central retinal artery through dangerous anastomosis from the MMA or AMA to the OphA. In addition, care should be taken to avoid reflux in the cavernous and petrosal branches of the MMA or AMA, as reflux may result in trigeminal or facial nerve palsy. Therefore, care should be taken to avoid reflux of the liquid embolic agent in the MMA to the foramen spinosum (1). When performing TAE via the meningeal branch of the OphA, there is a risk of occlusion of the central retinal artery. Because ILT and MHT of the ICA often act as feeders of sphenoidal region DAVF, during TAE via other feeders, a liquid embolic agent can go reflex into the cavernous ICA via the ILT or MHT. Therefore, the protective balloon in the ICA was helpful in preventing ICA occlusion. Because the toxicity of dimethyl sulfoxide can result in cardiac arrest due to the trigeminocardiac reflex, it was necessary to inject dimethyl sulfoxide slowly to prepare for cardiac resuscitation. Some complications, such as venous system occlusion, venous perforation, venous infarction, and intracranial hemorrhage, are associated with TVE.

## 9 Summary

EVT for sphenoidal region DAVFs is challenging. Curative EVT is needed to occlude fistulas and proximal venous drainage tubes. Various techniques can be used, including TAE, TVE, and direct puncture EVT. Among these methods, TAE was commonly used, and MMA or AMA can act as the gold standard. TVE should be limited in highly selective cases. As a last resort, sphenoidal region DAVFs can be embolized by direct puncture EVT. Complete EVT can lead to the obliteration of sphenoidal region DAVFs. However, these complications cannot be neglected.

## Author contributions

JY: Conceptualization, Writing – original draft, Writing – review & editing.
